# COVID-19 among medical personnel in the operating room

**DOI:** 10.1017/ice.2020.106

**Published:** 2020-04-03

**Authors:** Pathum Sookaromdee, Viroj Wiwanitkit

**Affiliations:** 1 TWS Medical Center, Bangkok Thailand; 2 Dr DY Patil University, Pune, India; 3 Hainan Medical University, Haikou, China; 4 Chulalongkorn University, Bangkok Thailand

*To the Editor*—Surgical infection is an important issue in hospital infection control; infection can occur in a patient receiving an operation. With the new coronavirus infection, COVID-19, there is a risk of nosocomial infection. Glauser^1^ proposed a protocol to keep COVID-19 out of hospitals. However, the infection might be carried by medical personnel. The issue of COVID-19 among medical personnel working in the operating room has not been well clarified.

Here, we provide reports from Thailand, a country with the second-most COVID-19 infections at one point in the worldwide outbreak timeline.^2^ As of March 27, 2020, there were 1,136 patients with COVID-19 in Thailand, and 2 of these were medical personnel working in operating rooms. These cases occurred in 2 different rural hospitals. The first case was an anesthesiologist and the second case was an internist working as a surgeon. These 2 patients had regular work rotations in operating rooms during the presymptomatic COVID-19 period. Surveillance for possible transmission to patients and other personnel is presently under way.

In fact, COVID-19 has been sporadically reported in a patients receiving surgery, causing special attention to be focused on management techniques related to patients.^3,4^ However, no reports have been published on COVID-19 among members of the medical teams who practice in operating rooms. Although surgical infection studies usually focus on patients, it is important to give attention to the practitioners who work in operation room as well.
